# Vulnerability of a top marine predator to coastal storms: a relationship between hydrodynamic drivers and stranding rates of newborn pinnipeds

**DOI:** 10.1038/s41598-020-69124-6

**Published:** 2020-07-30

**Authors:** M. Sepúlveda, R. A. Quiñones, C. Esparza, P. Carrasco, P. Winckler

**Affiliations:** 1 Centro de Investigación y Gestión de Recursos Naturales (CIGREN), Facultad de Ciencias, Universidad de Valparaíso, Valparaíso, Chile; 2 Núcleo Milenio de Salmónidos Invasores (INVASAL), Concepción, Chile; 3 Programa de Investigación Marina de Excelencia (PIMEX), Departamento de Oceanografía, Facultad de Ciencias Naturales y Oceanográficas, Universidad de Concepción, Concepción, Chile; 4 Centro de Investigación Para La Gestión Integrada del Riesgo de Desastres (CIGIDEN), Santiago, Chile; 5 Escuela de Ingeniería Civil Oceánica, Universidad de Valparaíso, Valparaíso, Chile; 6 Centro de Observación Marino Para Estudios de Riesgos del Ambiente Costero (COSTA-R), Universidad de Valparaíso, Valparaíso, Chile

**Keywords:** Ecology, Zoology, Ecology, Environmental sciences, Ocean sciences

## Abstract

Coastal storms have increased in recent decades, affecting many species, including the South American sea lion (*Otaria byronia*). Reports of stranded sea lion pups are becoming common in Chile, presumably due to the increase in the frequency and intensity of coastal storms. To validate this assumption, a 10-year database was built by coupling wave generation and coastal propagation models to correlate pure wave parameters (significant wave height Hs, peak period Tp, normalized wave power Hs^2^ Tp) and wave parameters including the tidal level (maximum surface elevation η, modified wave power η^2^ Tp) with records of stranded pups in Cobquecura, the largest breeding colony in central Chile. The correlation between the number of pups stranded per day and wave parameters in the first half of January and the last half of February is poor, while they are stronger for the second half of January and the first half of February. The higher number of stranded pups coincide with coastal storms with normalized wave power values exceeding a threshold of 100 m^2^/s. Conversely, below this threshold there is wide dispersion between the number of strandings and wave parameters. Identifying wave parameter thresholds could be used to predict when newborn pups will be most affected by coastal storms, and thus help institutions to develop remediation techniques for animals at risk.

## Introduction

Climate variability and change in the marine environment are emerging issues that have been reported to affect a wide range of species in different ways^1–4^. Signs of climate change include changes in air and sea surface temperatures, a rise in the absolute mean sea level, changes in salinity, ocean acidification, and increased frequency and intensity of extreme events, among others^5^. All of these signs are causing shifts in the abundance and distribution of several species, loss of habitat and changes in survival rates and breeding success. Some responses have been relatively consistent among species, such as a general advance in the timing of breeding and the migration of several bird species^6,7^. However, other responses, like population size and breeding success are less consistent, and vary by species and location^8,9^.

Most studies on climate change in marine environments have focused on the rise in temperature and changes in the availability of resources^10,11^. Other effects, such as the occurrence of coastal storms, have been overlooked, even though extreme events are expected to become more common over time, as they are associated with climate variability and global environment change^12^. A recent study showed that maximum monthly wave heights and the number of extreme events have both increased in central Chile in the last 60 years^13^. The majority of these events occur during the austral winter, but they sometimes occur in summer as a consequence of long swells generated in the north Pacific^13^. In general, the main focus of studies that have evaluated the impacts of coastal storms has been damage to infrastructure and urban areas^14^. The impacts on marine fauna that share coastal habitats with humans have received much less attention. In this context, it is relevant to know how extreme events affect coastal populations, and whether species can adapt to these changes^15^.

Iconic apex marine predators such as seabirds and marine mammals could be severely affected by variations and changes in the marine environment^8^. Storms affect marine mega fauna in different ways, such as habitat destruction and increased risk of stranding, with inshore species being particularly vulnerable^16^. Studies of shorebirds have demonstrated that increased storm intensity and frequency reduces the availability of habitats for nets establishment^17^. For pinnipeds (seals, sea lions and walrus), site selection is based on the proximity to favorable foraging areas and on the availability of terrain that allows access and egress during variable tidal heights^18^. For breeding colonies in particular, protection from wind and waves also plays a critical role in site selection, as pups have a better chance of survival in sheltered sites with favorable thermal protection^19^. Indeed, significant neonate mortality may result from unusual fluctuations in tidal height and storm surges because pups under three months of age cannot swim competently^20^. In Australia, Arnould & Littnan^21^ and Pemberton & Gales^22^ have reported that a considerable number of Australian fur seal (*Arctocephalus pusillus doriferus*) pups are left stranded when they are washed into the ocean by high swells and storms. However, to our knowledge no studies have monitored the effects of different intensities of coastal storms on pinniped species, with the identification of a threshold over which there are predictable effects on individuals and populations.

The South American sea lion (SASL, *Otaria byronia*) is an otariid species with a wide coastal distribution in both the Pacific and the Atlantic Oceans in South America^23^. This species can be found in several coastal rookeries along the Chilean coast, with a total abundance for the country of nearly 130,000 individuals^24,25^. It is classified as a Least Concern species under the International Union for Conservation of Nature’s system of categorizing species according to risk of extinction. In Chile, SASL is an important top predator in marine ecosystems, playing an important role in structuring the trophic relationships within food webs^26,27^. Like most otariids, the pupping season for the SASL is in January and February (austral summer season), with the mean peak period for parturition from mid-January to mid-February^28^. During this period, pups are weak and must remain in the breeding colony for at least one to two months, due to their low locomotory abilities at sea^29^. In this time of early life, pups can be washed out of the colony by waves, making them extremely vulnerable to stranding and starvation due to separation from their mothers^30^.

Considering an increase in the intensity of coastal storms in recent years, in this study we hypothesize that the number of stranded sea lion pups can be predicted by statistical wave parameters. To test this hypothesis, we compared the statistical parameters of coastal storms with the number of stranded SASL pups during the last decade in the largest breeding colony in central Chile.

## Results

### Number of stranded SASL pups

**Figure 1:**
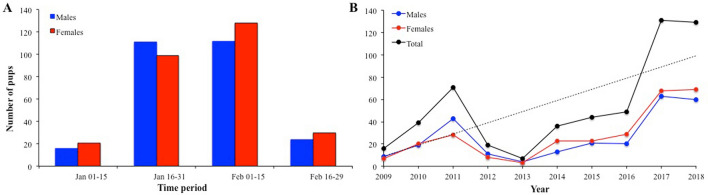
Number of stranded male and female pups that were captured adjacent to the Cobquecura colony **A** in different time periods from 2009 to 2018 (all years combined) and **B** aggregated yearly in the same period. A linear fit is included, showing an increase of nearly 10 pups per year.

A total of 541 stranded alive pups (i.e. pups swept away from the rookery and washed up on the beach), males and females, were counted and measured over a 10-year period (Table 1), most of them were registered between mid-January and mid-February (Fig. 1A). In general, the number of stranded pups increased over the 10-year period by around 10 pups per year (Fig. 1B). The percentage of total pups born in the colony that were stranded ranged from 0.7% (2013) to 11.6% (2018) (Table 1). Notably, the number of stranded pups represented more than 10% of those born in 2011, 2017 and 2018.

**Table 1:** Number of stranded South American sea lion (*Otaria byronia*) pups counted on the beach adjacent to the Cobquecura breeding colony from 2009 to 2018.

Year	Males	Females	Total	Proportion of the total pups in the colony (%)
2009	10	6	16	2.5
2010	19	20	39	7.5
2011	43	28	71	10.0
2012	11	8	19	2.2
2013	4	3	7	0.7
2014	13	23	36	4.7
2015	20	24	44	4.2
2016	20	29	49	4.8
2017	63	68	131	10.5
2018	60	69	129	11.6
Total	263	278	541	5.9 ± 3.8

Supplementary Table S1 gives the length and weight statistics obtained for male and female pups during each fortnight, along with the estimated parameters of the length–weight ratio and the coefficient of determination *r*^2^. All regressions for fortnights 2 and 3 were highly significant, although coefficients of determination were modest, indicating considerable unexplained variation. No relationships between length and weight were found for pups sampled during fortnights 1 and 4 (Supplementary Table S1).

### Wave climate

**Figure 2:**
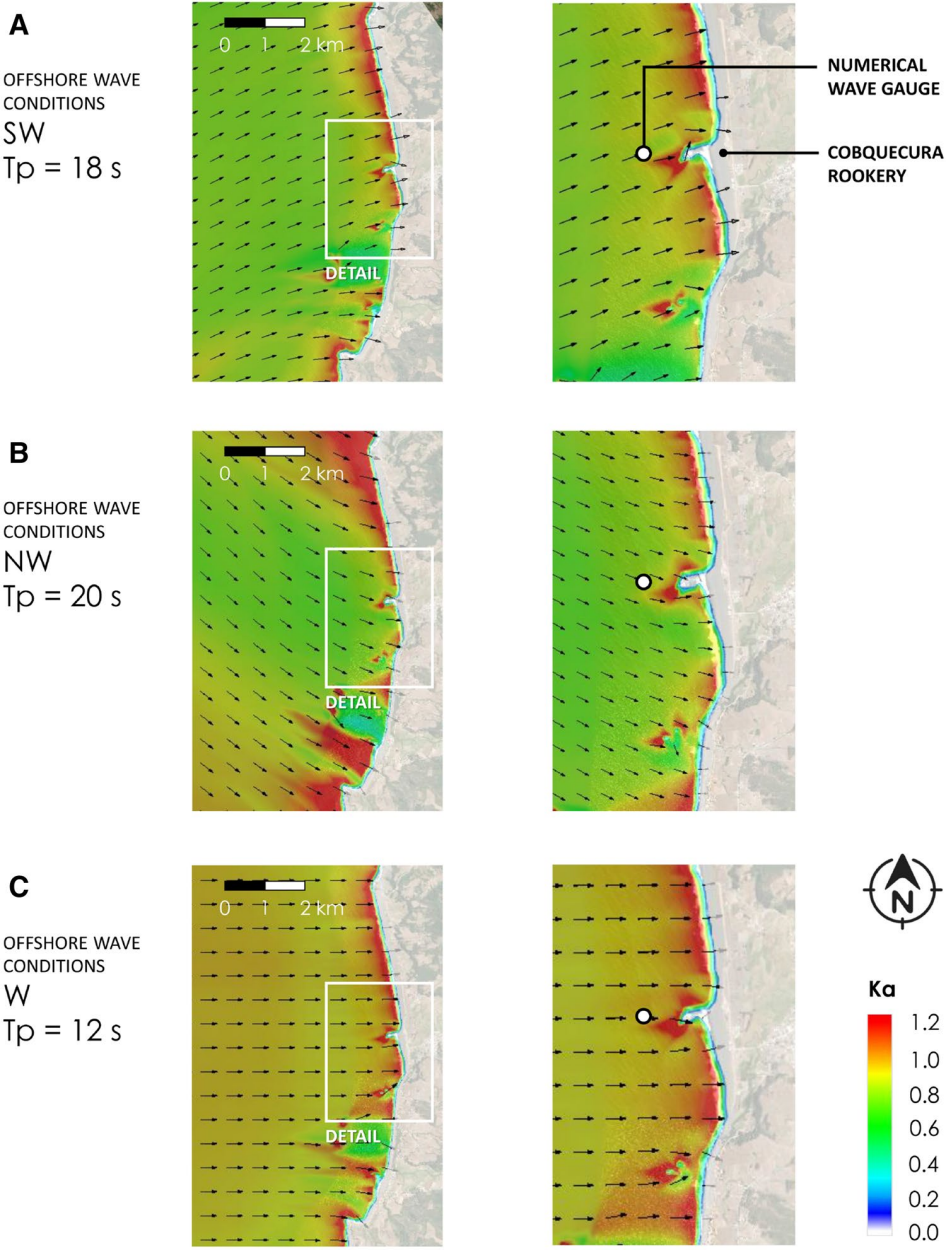
Wave agitation coefficient (Ka) for a representative selection of nearshore wave patterns during coastal storms, selected from 96 offshore wave conditions in the vicinity of the Cobquecura colony. **A** SW swell with a peak period of Tp = 18 s generated in the southeastern Pacific; **B** NW Aleutian swell with Tp = 20 s engendered in the north Pacific, and **C** W swell with Tp = 12 s generated off the coast of Cobquecura.

Figure 2 shows the wave climate in the nearshore numerical wave gauge at 10 m depth. The coastal wave patterns associated with a distant Southwestern (SW) swell of the peak period (Tp) = 12 s (Fig. 2A) shows that for this sea state, waves are strongly refracted regardless of the offshore wave period and direction as a consequence of the relatively shallow and smooth bathymetry formed by sands drifting from the south. However, wave height is strongly amplified near the site, with the agitation coefficient (Ka) ~ 1.2, due to the combined effect of shoaling and refraction. Figure 2B shows coastal wave patterns associated with an Aleutian swell of Tp = 20 s. As for the SW swell (and for most analyzed cases), waves shoal and refract near the shore, with wave height decreasing slightly off the coast of Cobquecura, but significantly, increasing in the vicinity of the rookery. Figure 2C shows a Western (W) swell of Tp = 12 s generated in mid-latitudes (~ 34°S–38°S) near South America. In this case, refraction is less significant and shoaling explains the increase in wave height near the rookery. The above examples, chosen from a total of 96 cases, provide a representative selection of near shore wave patterns during coastal storms.

In statistical terms (Supplementary Table S2), bivariate wave statistics for the January–February (JF) period in the numerical node at 10 m depth offshore Cobquecura colony show that 78.1% of the sea states have directions between 240° and 270° (i.e. with wave patterns similar to those in Fig. 2A, C) and that 71.5% of the significant wave heights are between 1.5 and 2.5 m. About 73% of waves are in the range between 10 and 16 s, while 10.1% exceed 14 s. On the other hand, Aleutian swells (Fig. 2B) account for 19.4%, with only 6% of wave heights above 2 m. Wave heights are seldom below 1 m.

### Correlation between coastal storms and number of pups stranded on the beach

**Figure 3:**
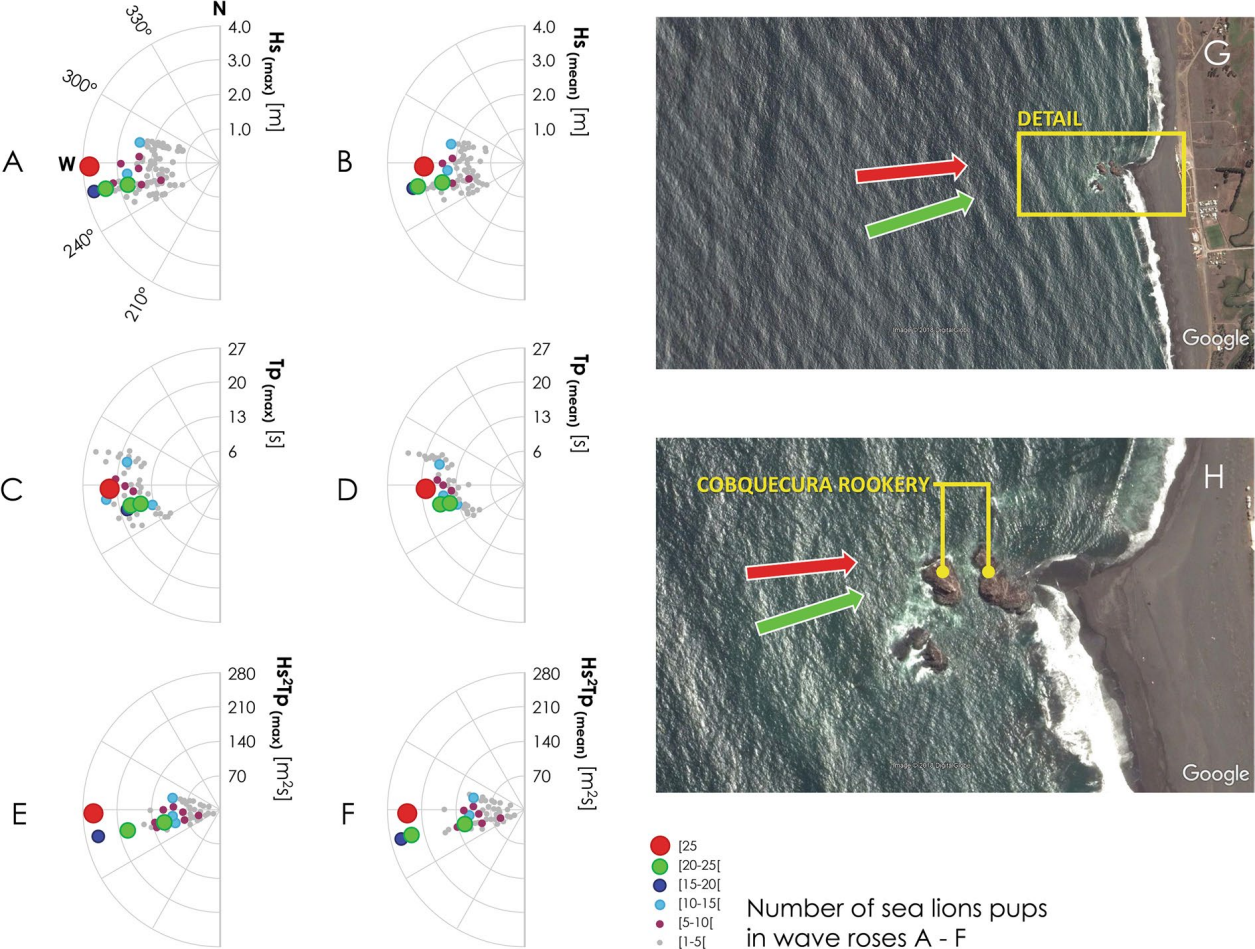
Wave roses for the January–February period between 2009 and 2018 in the vicinity of the Cobquecura colony for **A** the maximum daily significant wave height Hs_(max)_, **B** the mean daily significant wave height Hs_(mean)_, **C** the maximum daily peak period Tp_(max)_, **D** the mean daily peak period Tp_(mean)_, **E** the maximum daily value of wave power Hs^2^ Tp_(max)_, **F** the mean daily value of wave power Hs^2^ Tp_(mean)_, **G** a satellite view of the wave pattern in the coastal area, and **H** zoom-ins of the Cobquecura rookery for W (red) and WSW (green) swells shown in the wave roses. The number of sea lion pups found on the beach per day is shown in different colors and sizes.

Figure 3A–F show sea states in wave roses for the JF period in the vicinity of Cobquecura colony, expressed in terms of some of the statistical wave parameters (e.g. wave height (Hs), peak period (Tp), wave power (Hs^2^ Tp)) together with the number of SASL pups found on the beach per day. Figure 3G, H show the local wave direction in relation to the Cobquecura rookery for W and Western-southwestern (WSW) swells in the wave roses. An example of how wave parameters are related to the number of pups stranded on the beach is the intense coastal storm of January 30 and 31 2011 (Supplementary Fig. S1). Massive strandings of pups occurred in Cobquecura colony after sea states with heights of Hs = 3.8 m, periods beyond Tp = 20 s and nearly W waves affected the area. This storm is represented by the red dot in Fig. 3A–F and the red arrow in Fig. 3G, H. These unusual conditions were reflected in one of the most powerful coastal storms throughout the dataset, with Hs^2^ Tp above 250 m^2^s. While the maximum statistical surface elevation (η) usually ranges between η =  + 2 and + 3 m LAT, it reached a maximum of η =  + 4.5 m LAT on January 30.

**Figure 4:**
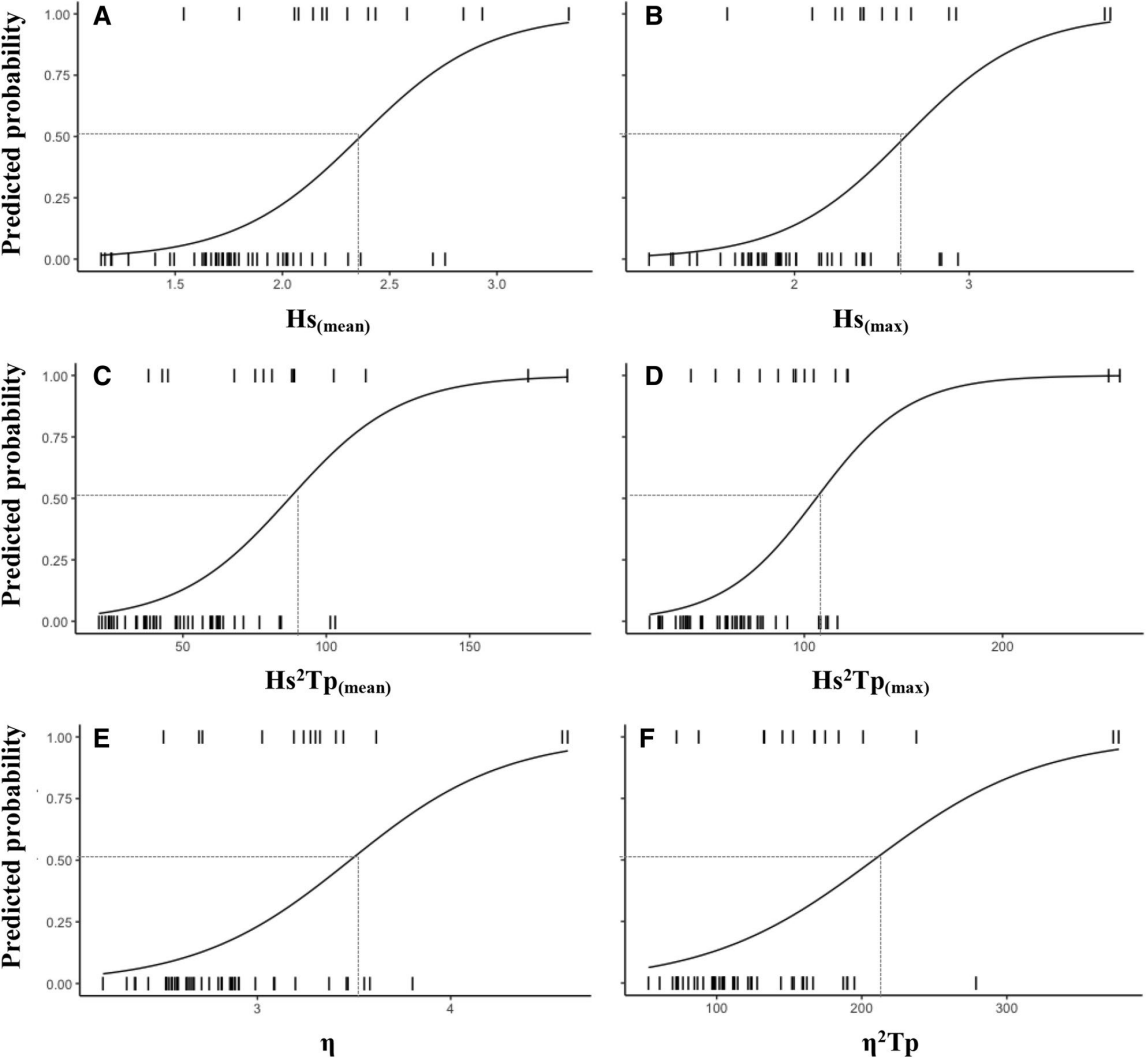
Binomial logistic regression predicted probabilities when newborn pups will be most affected by coastal storms during the Fortnight 2 (16–31 January), as a function of **A** the mean daily significant wave height Hs_(mean)_, **B** the maximum daily significant wave height Hs_(max)_, **C** the mean daily value of wave power Hs^2^Tp _(mean)_, **D** the maximum daily value of wave power Hs^2^ Tp _(max)_, **E** the maximum elevation η, **F** the modified wave power including the tide η^2^ Tp. Dashed lines represent the threshold for a probability of 50%.

**Figure 5:**
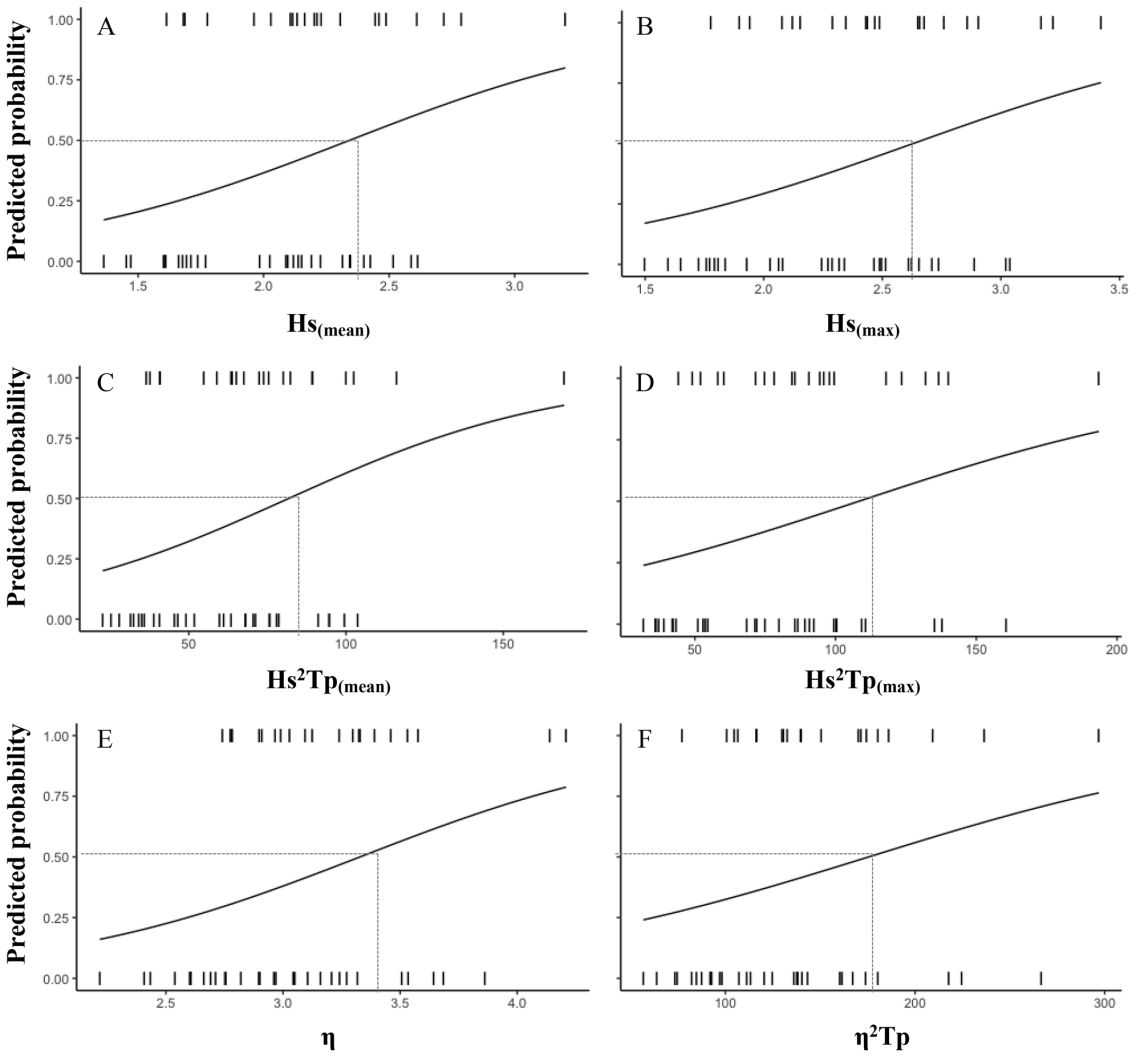
Binomial logistic regression predicted probabilities when newborn pups will be most affected by coastal storms during the Fortnight 3 (01–15 February), as a function of **A** the mean daily significant wave height Hs_(mean)_, **B** the maximum daily significant wave height Hs_(max)_, **C** the mean daily value of wave power Hs^2^ Tp _(mean)_, **D** the maximum daily value of wave power Hs^2^ Tp _(max)_, **E** the maximum elevation η, **F** the modified wave power including the tide η^2^ Tp. Dashed lines represent the threshold for a probability of 50%.

Table 2 shows the relationship between the number of stranded pups on the beach and the statistical wave parameters for the four fortnight intervals from 2009 to 2018. It is noted that the correlations for all the data during the first and last fortnights (January 1–15 and February 16–29) were very low; no significant correlations were found for any of the parameters analyzed. Correlations dramatically increased when the analysis was restricted to the second and third fortnights (January 16–31 and February 1–15) (Supplementary Fig. S2). During the second fortnight, the proxies of Hs^2^ Tp showed the best performance, followed by the Hs_(max)_, the η and the modified wave power including the tide (η^2^ Tp) (Table 2). The proxies of Hs^2^ Tp also showed the best performance during the third fortnight. Binomial logistic regression indicated that during the second and third fortnights thresholds could be defined for those wave parameters (Figs. 4, 5). In particular, the 50% probability of exceeding three stranded pups is predicted to be higher with a threshold of Hs^2^ Tp  ~ 10 m^2^/s during the second fortnight (Fig. 4).

**Table 2:** Regressions between the number of pups stranded on the beach adjacent to the Cobquecura breeding colony and statistical wave parameters for each sea state at fortnight intervals in the January–February period between 2009 and 2018.

Parameter	01–15 January		16–31 January		01–15 February		16–29 February	
	F	R^2^	F	R^2^	F	R^2^	F	**R**^2^
Hs_(mean)_	1.52	0.13	22.04	0.45***	13.58	0.36***	0.18	0.09
Hs_(max)_	1.37	0.11	60.19	0.69***	10.08	0.30***	3.79	0.26*
Tp_(mean)_	0.02	0.001	1.31	0.05	3.19	0.05*	0.84	0.07
Tp_(max)_	0.26	0.02	1.43	0.05	2.90	0.11	0.56	0.05
Hs^2^ Tp_(mean)_	0.91	0.08	59.38	0.69***	11.99	0.33***	1.28	0.11
Hs^2^ Tp_(max)_	0.92	0.08	94.89	0.77***	7.45	0.24**	1.05	0.09
η	0.27	0.02	59.00	0.67***	10.87	0.31***	1.10	0.09
η^2^ Tp	0.06	0.006	53.35	0.66***	6.56	0.21**	0.36	0.03

Hs_(mean)_: the mean daily significant wave height; Hs_(max)_: the maximum daily significant wave height; Tp_(mean)_: the mean daily peak period; Tp_(max)_: the maximum daily peak period; Hs^2^ Tp_(mean)_: the mean daily value of wave power; Hs^2^ Tp_(max)_: the maximum daily value of wave power; η: maximum surface elevation; η^2^ Tp: modified wave power including the tide. Significant differences are highlighted in bold.

*P < 0.05, **P < 0.01, ***P < 0.001.

## Discussion

The impact of climate change on terrestrial mammals has been extensively examined in the literature. However, how marine mammals react to climate change, and specifically to the increase in the occurrence and intensity of coastal storms, have been seldom analyzed. The evidence indicates that rare extreme climatic events, like coastal storms, have become much more frequent and intense in the last decade, especially during the summer when SASL females give birth to their pups. Although different studies have noted that coastal storms affect pinniped pup survival^22,31^, to our knowledge this is the first study that assesses the effects of wave parameters on such phenomena. We discuss here how the increase in the number of pup strandings may affect population growth and the responses of SASL, and how this should be considered in management policies concerning this species in Chile. Although this study focuses on one species and one study area, climate change over the next 100 years will lead to increases in sea levels and the severity of storm surges in many places^30^, which in turn may affect different pinniped species around the world.

The analysis shows that correlations between statistical wave parameters and stranded pups on the beach neighboring the Cobquecura breeding colony is best predicted by Hs^2^ Tp and η^2^ Tp. As a reference, for a range of values of Hs^2^ Tp _max_ from 32 to 140 m^2^/s, the number of stranded pups fluctuates between 1 and 23 per day during the third fortnight (Supplementary Fig. S2(C)). The average overtopping discharge scales with a power (~ 1) of both the significant wave height and the mean wave period (which also scales with the peak period), and is very sensitive to the tide level. Thus, the value of η^2^ Tp is determined by the same variables used to quantify the wave overtopping discharge, which is apparently the mechanism by which pups are washed away from the rookery. Individual parameters (i.e. Hs and Tp) showed a lower correlation with the number of pups washed away.

It is evident that the swells that affect pups are restricted to a band of 50° (from 240° to 290°), while events that cause significant casualties have directions between 255° and 270°, in which the shelter of the southwest rock on the colony is negligible and wave overtopping is favored. The relatively narrow directional range of waves impinging the rookeries is explained by the strong refraction that waves undergo in the shoaling region offshore the rookeries. The southwest rookery is only an effective shelter against SSW to SW waves, which account for 40% of waves in the JF period (225° ≤ Dpk ≤ 255°); while the rest of the time (60%) waves impact the rookeries where pups are almost directly exposed.

In general, the correlation between wave parameters and the number of pups stranded is notably stronger for the second half of January and the first half of February. This positive relationship may be because the number of SASL pups born in a colony increases as the month of January progresses, reaching a peak in the second half of January and the first half of February^28^. Thus, the probability of a pup being swept away from the rookery and washed up on the beach increases. As well, SASL mothers spend less time on shore as pup get older^32–34^, so pups are left alone for longer periods, making them more vulnerable to coastal storms. The low correlation between wave parameters and the number of stranded pups in the first fortnight of January may be because the number of newborn pups in the colony during this period is still very low. Similarly, the lack of any relationship between wave parameters and the number of stranded pups in the last two weeks of February could be because pups are bigger and heavier, and thus able to swim back to the colony by themselves^29^, which avoids animals being washed up on the adjacent beach.

Our results show that the number of pups stranded on the beach adjacent to the colony was especially high in certain years, exceeding 10% of total pup abundance in the colony in those years. Given that female SASL give birth to a single pup each year, a higher frequency of coastal storms that coincide with the breeding season can dramatically affect pups^30^, and ultimately may have a serious impact on the population. This in turn can impact juvenile recruitment and reduce the likelihood of recovery between events, thereby reducing reproductive output and ultimately causing population decline^15,30^. Long-term abundance monitoring, together with the estimation of demographic parameters of this population, such as age/class mortality and fecundity, provides critical information for SASL conservation and management. However, there presently are insufficient demographic data to reliably estimate the effects of pup mortality due to storm frequency and intensity on the SASL population dynamics in Cobquecura. We recommend that future studies focus on determining age-structured dynamics of the SASL population at Cobquecura in order to put the impact of pup strandings into context.

The number of stranded pups has varied greatly over the years. In some years (e.g. 2013), the number and proportion of the total abundance was very low (0.7%), while in other years it was notably high (> 10% in 2011, 2017 and 2018). Similar results were found with Australian fur seal pups, where major fluctuations in pup mortality have been associated with large storm surge events^22^. The number of pups in a colony can be underestimated given that not all the pups are visible, and that not all pups have been born by the time of an aerial survey. Consequently, estimates should be taken with caution. We also recognize that our results are based on a particular SASL colony, and thus the effects of statistical wave parameters on other colonies depend on other factors as well, such as coastal orientation, storm duration, position, bathymetry, topography and the elevation of the colony^30^. However, the approach used in this study could be used as a baseline to characterize wave parameters which, together with coastal early warning systems^35^, would be a useful tool for the conservation and management of coastal species that are affected, or could be affected, by coastal storms in the near future, such as birds^17^ and pinnipeds^21,22^.

In the face of the increasing severity of storm surges, individuals in colonies may respond by either: (1) continuing to breed in areas that are becoming increasingly inundated by coastal storms, potentially causing higher pup mortality rates and affecting the colony's long-term demographics; (2) retreating to areas that are higher or further from the coast to avoid waves, leading to higher operational breeding densities; and (3) moving to other colonies or colonizing new areas. Many pinniped species, including the SASL, display a high degree of natal site philopatry^36–38^. The Cobquecura and other colonies in Chile have been used consistently as they afford the best habitats for pup survival, providing access to water for thermoregulatory requirements and close proximity to suitable feeding areas. These attributes suggest that SASL moving to other colonies will not happen easily or rapidly.

Moving to other colonies or colonizing new areas is another possibility for the species in order to reduce pup mortality. Grandi et al.^37^ found a proliferation of new SASL colonies and the transformation of some haul-outs to breeding sites over time in northern Patagonia, Argentina, as a response to population growth. A similar expansion to new breeding colonies has been reported for other otariid pinnipeds, such as Subantarctic fur seals (*Arctocephalus tropicalis*) [39], Antarctic fur seals (*A. gazella*) [39], and Steller sea lions (*Eumetopias jubatus*)^40^. However, dispersal to other established sites or the establishment of new breeding colonies can in turn lead to an increase in colony densities, which is associated with increased pup mortality^41^. SASL expand to other colonies that are close to the focal one^37^. In this sense, if the new area does not offer much protection against coastal storms, moving to other colonies nearby may not be an improvement.

The results of our study demonstrate that negative impacts of coastal storms occur during the most vulnerable phase of pups’ lives. Even though we still cannot estimate the consequences of coastal storms on the future of the SASL, the ability of this species to adapt to the impact of climate change may be complicated by their being under various constraints, such as an accelerated rate of climate change^42^ and limited availability of alternative habitats owing to human coastal activity^43^. As the impact of climate change increases and the capacity of populations to recover lessens, more active intervention strategies may be necessary. For instance, the thresholds identified for some statistical wave parameters could be used to predict when newborn pups will be most affected by coastal storms. Local government and other agencies could use this information to improve their efforts to rescue and release pups. The feasibility and success of these management measures go beyond the subject of this study, but there is some evidence that these measures enhance the survival of stranded newborn pups following rescue and release^44^. Identifying statistical wave parameter thresholds for colonies of at-risk species can help institutions develop remediation techniques for animals at risk.

In conclusion, the results of this study demonstrate that the SASL is affected by extreme weather events that have a negative effect on pinniped wellbeing^45^. The accuracy of inputs such as coastal data points and topography should be improved and modeling techniques should be enhanced to include the influence of predicted increases in both the frequency and intensity of weather events. Understanding these impacts is crucial for the future management of this top marine predator.

## Materials and methods

### Study area

**Figure 6:**
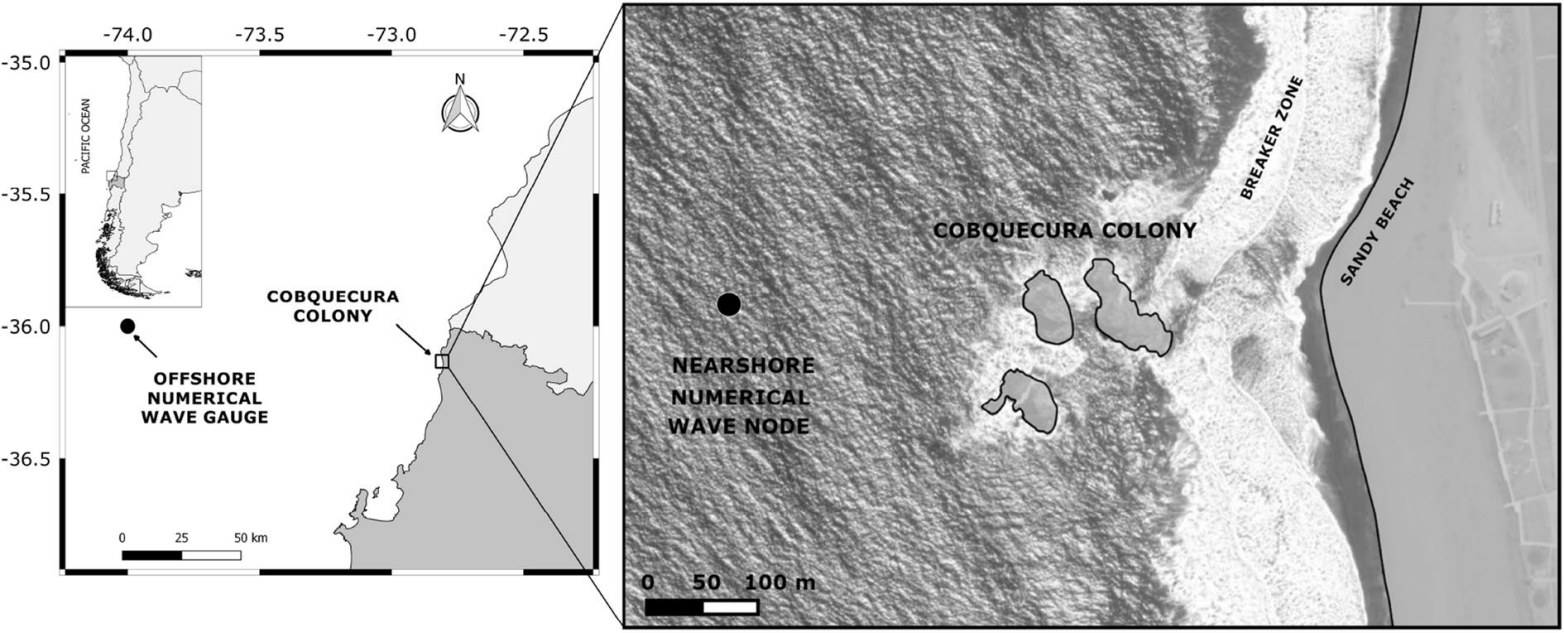
Geographic location of the South American sea lion (*Otaria byronia*) Cobquecura breeding colony on the coast of central Chile.

The breeding colony of Cobquecura (36° 07′ S, 72° 48′ W) is located approximately 80–100 m from the coast, in front of a long and open continental sandy beach on the coast of central Chile (Fig. 6), with high exposure to wind/wave action. Cobquecura is the largest breeding colony along the coast of central Chile, with approximately 3,200 animals, which represent ca. 20% of total abundance, and produces ~ 51% of the total pups born in central Chile^25^.

### Number of stranded SASL pups

During the breeding season, high waves frequently sweep away healthy pups from the colony and transport them alive to the adjacent continental beach. Considering this, sampling was conducted annually during the breeding seasons for a period of ten years from 2009 to 2018, between January 1 and February 29 to count the total number of pups that were stranded daily. The colony and the adjacent beach were monitored every day by one to three observers, who were placed in the beach adjacent to the colony from 09:00 AM to 6:00 PM. If a pup was found before 9:00 AM, it was assumed it had been stranded during the night and consequently it was counted as stranded in the same day. Most pups (> 90%) are stranded alive. We decided not to include dead pups in this study because in most cases it is not possible to know the date and cause of death. No other breeding colonies are close to the Cobquecura colony, so it was assumed that all stranded pups came from this colony.

Every year, stranded pups were rescued and retained by volunteers from a local animal welfare organization (Codeff) who let the pups rest safe from tourists. Once the pups appeared to be in better condition and the weather improved, the volunteers released the animals individually near the colony so they could return to it. Before releasing pups, researchers from the Program of Marine Research of Excellence (PIMEX; University of Concepción) identified them by sex and registered their standard length (L, straight line nose to tail, dorsal view) to the nearest 0.01 m, and body mass (M) to the nearest 0.1 kg. Before they were set free, the pups were marked with water-resistant paint on a front flipper nail to avoid measuring the same individuals again. All applicable national guidelines and regulations for the care and use of animals were followed, and animal rescues were approved by the National Service of Fisheries and Aquaculture (Sernapesca; Ministry of Economy, Chilean Government).

Four to five aerial surveys were conducted annually (1 or 2 in January, 2 in February and 1 in March) to estimate the percentage of pups born in the colony that were stranded every summer. From 2009 to 2011, the aerial surveys were carried out with a Cessna 172 airplane at an altitude of 70–100 m and at a speed of 100–150 km h^−1^. From 2012 to 2018, the surveys were conducted with Phantom Vision and Phantom 4 Advance drones equipped with a 12–20 megapixel wide-field-of-view camera (lens: 5–9 mm) and remotely controlled by an experienced pilot. Photographs covered the entire colony and were taken at an altitude of 25–30 m. Previous surveys at the colony indicated that SASL are not disturbed by flights at this altitude. Flights at similar or even lower altitudes do not appear to have affected pinniped behavior^46,47^. Photographs were vertical when possible, or with some angle in areas dominated by steep cliffs. Due to the irregular topography of the colony, the pilot manually regulated the diaphragm aperture of the photographs to match the desired exposure.

Three independent observers counted the number of sea lions from the photographic records using Photoshop software (Adobe Photoshop CS5.1, Version 12.1). Photographs were sequential and slightly overlapped to guarantee complete coverage of the colony. Photographic mosaics were created from multiple digital images to avoid double counting. Final values were estimated by averaging the counts from the 3 observers, with a maximum error of 10% among them. Every year, we considered the census with the highest abundance of pups to compare with the number of stranded pups annually.

### Storm characterization

In order to correlate the number of stranded pups with coastal storms, wave climate offshore from Cobquecura was assembled with the third-generation spectral model Wavewatch III v.4.18^48^ using the parameterization proposed by Ardhuin et al.^49^. Following Stopa & Cheung^50^ recommendations, we used wind fields at 10 m above mean sea level and ice coverage available from the Climate Forecast System Reanalysis CFSR^51^ to force the wave model. We used the ETOPO v.2^52^ global bathymetry, with 1° × 1° spatial resolution on a Pacific-wide domain. The shoreline, islands and rock formations were obtained from the Global Self-consistent, Hierarchical, High-resolution Geography Database (GSHHS), with a resolution of 40 m^53^. The model was calibrated following the steps proposed by Beyá et al.^54^. Hourly wave spectral data was obtained from January 1, 1991 to December 31, 2018 from an offshore numerical node at 36°S 74°W (Fig. 6), but the analysis was restricted to the JF period when strandings were recorded.

Wave data was then transferred to the coast by means of the Steady-state spectral WAVE model STWAVE v. 6.0^55^, a second-generation spectral wave model capable of simulating near shore processes. The digital elevation model was constructed from nautical charts from the Hydrographic and Oceanographic Service of the Chilean Navy^56^. Transfer functions built from synthetic wave spectra with significant wave heights of 1 m were used to transform wave data at the offshore numerical node to the site. A total of 96 representative offshore JONSWAP spectra were used^57^, covering periods between 4 and 26 s, every 2 s, with directions between 202.5° and 360°, every 22.5°. The propagation pattern was expressed in terms of the agitation coefficient (Ka), defined herein as the ratio between the significant wave height in the numerical node immediately offshore from Cobquecura and its value in deep waters, where it is unaffected by local conditions. The agitation coefficient represents the amplification (Ka > 1) or reduction (Ka < 1) of the significant wave height at the numerical node immediately offshore from Cobquecura, when compared to offshore conditions where waves are not perturbed by the seafloor.

**Figure 7:**
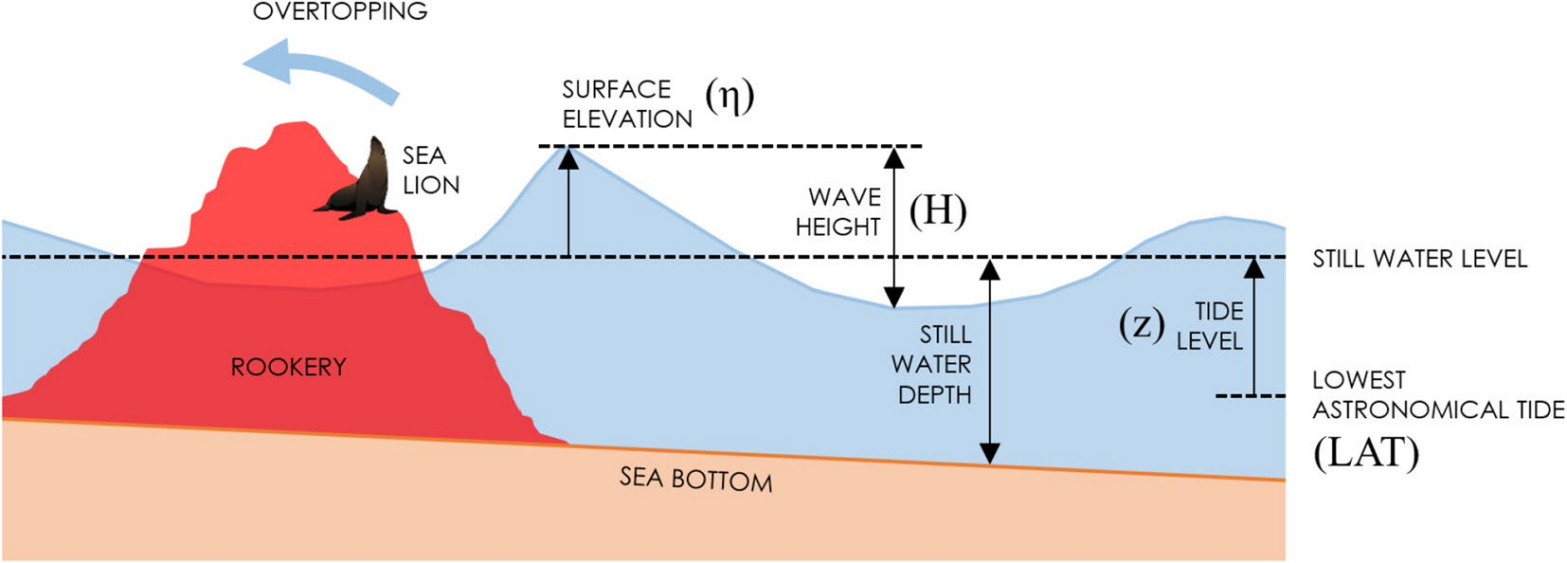
Relevant parameters used in the description of the local geometry and waves.

The astronomical tide level (z) was also included to better describe the effects of oceanographic conditions in the breeding colony (Fig. 7). Harmonic analysis using T-Tide was carried out to reconstruct the astronomical tide for the analyzed period^58^. Hourly sea level records were obtained at Constitución (35°19′ S, 72°24′ W), located 100 km north of Cobquecura (due to the relatively straight coastline and the absence of nearshore geographical features, the spatial gradient of the tide is negligible between the two sites).

### Coastal storms and the number of stranded pups

A series of modeled statistical wave parameters for each sea state in the numerical node immediately offshore from Cobquecura were correlated with the number of pups found on the beach on a daily basis during the JF period. The definition of relevant parameters used in the description of the local geometry and waves is included in Fig. 7. These parameters were the significant wave height Hs (measured in m), defined as the average of wave heights (H, as defined in Fig. 7) of the one-third highest waves, the peak period Tp (measured in s) is the period at which the wave energy spectrum is maximum, and the normalized wave power Hs^2^ Tp (measured in m^2^ s), which scales with the wave energy flux P = (ρg/64π) Hs^2^ Tp in deep waters^59^. Note that the significant wave height Hs is obtained from the spectrum assuming Hm_0_ = 4(m_0_)^1/2^, where m_0_ is the zeroth order momentum of the spectrum and Hm_0_ denotes the significant wave height determined from the spectrum^58^, and further assuming Hs = Hm_0_. While Hs is determined from time-domain analysis, the two quantities are equal when wave height follows the Rayleigh distribution^60^.

The analysis was enriched by the addition of the tidal effect of local wave parameters. Once the tide had been computed for the entire historical period, we estimated the maximum statistical surface elevation with respect to the Lowest Astronomical Tide LAT η (measured in m) at a local depth of 10 m (Fig. 2). Like Hs, η provides a statistical measure of the height of the waves, but it also implicitly includes the astronomical tide with respect to the LAT. The parameter η^2^ Tp (measured in m^2^ s), referred to hereafter as the modified wave power including the tide, was also tested. This parameter also scales with the wave energy flux (as does Hs^2^ Tp), but additionally includes the tide level in its computation. The aforementioned statistical wave parameters (Hs, Tp, Hs^2^ Tp, η, η^2^ Tp) were computed for the maximum sea state of the day -between 0:00 AM to 12:00 PM- and for the mean value averaged on the day when stranded pups were counted.

### Statistical analysis

The relationship between the number of stranded pups and the wave parameters were analyzed using second order polynomial regressions. Standard and body mass were log-transformed and the linearized relationship fitted by least-squares regression. Analyses were done separately for the two fortnights of January and the two of February, since the abundance and behavior of pups varies over the course of the breeding season. Wave parameter variables that were significant (α = 0.05) in the least-squares regression were analyzed to define thresholds that can be used to predict when newborn pups will be most affected by coastal storms. For each variable, a binomial logistic regression model was performed, defined as 0 (stranding events with ≤ 3 pups) and 1 (stranding events with > 3 pups). This criterion was based on the break in the frequency histogram for the number of stranded pups for each event. Each threshold value was defined with a predicted probability of 50%, using a similar approach of LD_50_ (the dose where historically 50% of the animals are predicted to die) analysis. All analyses were conducted using R Studio^61^.

## Supplementary information

Supplementary file1 (DOCX 1347 kb)

Supplementary file2 (DOCX 195 kb)

Supplementary file3 (DOCX 96 kb)

Supplementary file4 (DOCX 107 kb)
