# Supplementary material for: Vulnerability of a top marine predator to coastal storms: a relationship between hydrodynamic drivers and stranding rates of newborn pinnipeds
Source: Sci Rep. 2020 Jul 30;10:12807. doi: 10.1038/s41598-020-69124-6 (PMC7393492; doi:10.1038/s41598-020-69124-6)

**VULNERABILITY OF A TOP MARINE PREDATOR TO COASTAL STORMS: A RELATIONSHIP BETWEEN HYDRODYNAMIC DRIVERS AND STRANDING RATES OF NEWBORN PINNIPEDS**

Sepúlveda M.^1,2,3,*^, R. Quiñones^3,4^, C. Esparza^5^, P. Carrasco^3,4^ & P. Winckler^5,6,7^

^1^Centro de Investigación y Gestión de los Recursos Naturales (CIGREN), Universidad de Valparaíso, Valparaíso, Chile

^2^Núcleo Milenio de Salmónidos Invasores (INVASAL)

^3^Programa de Investigación Marina de Excelencia (PIMEX), Departamento de Oceanografía, Facultad de Ciencias Naturales y Oceanográficas, Casilla 160-C, Universidad de Concepción, Casilla 160-C, Concepción, Chile

^4^Interdisciplinary Center for Aquaculture Research (INCAR-FONDAP), Universidad de Concepción, O’Higgins 1695, Concepción 4070007, Chile

^5^Escuela de Ingeniería Civil Oceánica, Universidad de Valparaíso, Valparaíso, Chile

^6^Centro de Investigación para la Gestión Integrada del Riesgo de Desastres (CIGIDEN), Chile.

^7^Centro de Observación Marino para estudios de Riesgos del Ambiente Costero (COSTAR), Chile

*Corresponding author: Gran Bretaña 1111, Playa Ancha, Valparaíso, Chile. E-mail: [maritza.sepulveda@uv.cl](mailto:maritza.sepulveda@uv.cl). Phone: +56322508346

**SUPPLEMENTARY MATERIAL**

Figure S1. Relationship between the number of pups stranded on the beach and the wave parameters during 2011. A) Hs: the daily significant wave height Fortnight, B) Tp: the daily peak period, C) Hs^2^Tp: the daily value of wave power, D) η: maximum surface elevation, and E) η^2^Tp: modified wave power including the tide.


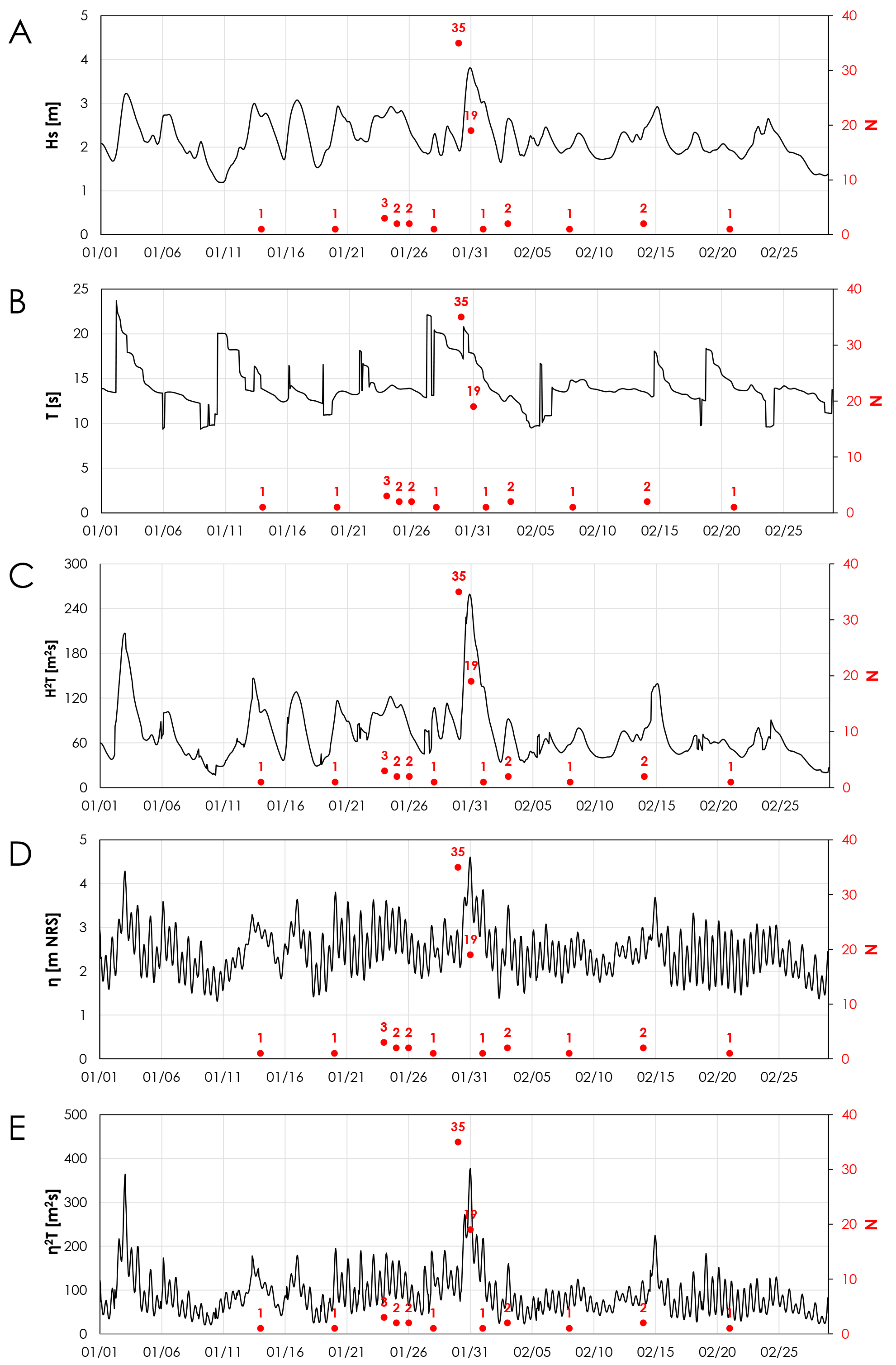

Supplement: Supplementary file 1 — Supplementary file1 (DOCX 1347 kb) [file 41598_2020_69124_MOESM1_ESM.docx]
