# Supplementary material for: Vulnerability of a top marine predator to coastal storms: a relationship between hydrodynamic drivers and stranding rates of newborn pinnipeds
Source: Sci Rep. 2020 Jul 30;10:12807. doi: 10.1038/s41598-020-69124-6 (PMC7393492; doi:10.1038/s41598-020-69124-6)
Supplement: Supplementary file 3 — Supplementary file3 (DOCX 96 kb) [file 41598_2020_69124_MOESM3_ESM.docx]

**VULNERABILITY OF A TOP MARINE PREDATOR TO COASTAL STORMS: A RELATIONSHIP BETWEEN HYDRODYNAMIC DRIVERS AND STRANDING RATES OF NEWBORN PINNIPEDS**

Sepúlveda M.^1,2,3,*^, R. Quiñones^3,4^, C. Esparza^5^, P. Carrasco^3,4^ & P. Winckler^5,6,7^

^1^Centro de Investigación y Gestión de los Recursos Naturales (CIGREN), Universidad de Valparaíso, Valparaíso, Chile

^2^Núcleo Milenio de Salmónidos Invasores (INVASAL)

^3^Programa de Investigación Marina de Excelencia (PIMEX), Departamento de Oceanografía, Facultad de Ciencias Naturales y Oceanográficas, Casilla 160-C, Universidad de Concepción, Casilla 160-C, Concepción, Chile

^4^Interdisciplinary Center for Aquaculture Research (INCAR-FONDAP), Universidad de Concepción, O’Higgins 1695, Concepción 4070007, Chile

^5^Escuela de Ingeniería Civil Oceánica, Universidad de Valparaíso, Valparaíso, Chile

^6^Centro de Investigación para la Gestión Integrada del Riesgo de Desastres (CIGIDEN), Chile.

^7^Centro de Observación Marino para estudios de Riesgos del Ambiente Costero (COSTAR), Chile

*Corresponding author: Gran Bretaña 1111, Playa Ancha, Valparaíso, Chile. E-mail: [maritza.sepulveda@uv.cl](mailto:maritza.sepulveda@uv.cl). Phone: +56322508346

**SUPPLEMENTARY MATERIAL**

Table S1. Weight-length ratios for South American sea lion pups stranded on the beach adjacent to the Cobquecura breeding colony at fortnight intervals in the January-February period between 2009 and 2018. S.D.: Standard deviation. Significant differences are highlighted in bold. Fortnight 1: 01-15 January; Fortnight 2: 16-31 January; Fortnight 3: 01-15 February; Fortnight 4: 16-31 February

| **Fortnight** | **Sex** | ***n*** | **Weight (kg)** | | **Length (cm)** | | **Parameters of the ratio** | | | ***P*** |
| --- | --- | --- | --- | --- | --- | --- | --- | --- | --- | --- |
|  |  |  | **Mean** | **S.D.** | **Mean** | **S.D.** | ***a*** | ***b*** | ***r^2^*** |  |
| 1 | Males | 16 | 12.1 | 1.6 | 76.6 | 4.2 | 0.2683 | 0.5738 | 0.0131 | 0.6728 |
|  | Females | 22 | 10.6 | 1.5 | 78.3 | 4.8 | 0.0880 | 0.8517 | 0.0014 | 0.8698 |
|  | Both | 38 | 11.2 | 1.7 | 77.6 | 4.6 | -0.0512 | 1.1414 | 0.0003 | 0.9067 |
| 2 | Males | 111 | 13.0 | 1.9 | 80.0 | 4.8 | 0.8288 | -0.4676 | 0.1059 | **0.0005** |
|  | Females | 95 | 12.0 | 1.7 | 78.1 | 5.4 | 0.7161 | -0.2816 | 0.1067 | **0.0012** |
|  | Both | 206 | 12.5 | 1.9 | 79.1 | 5.1 | 0.8705 | -0.5594 | 0.1308 | **<0.0001** |
| 3 | Males | 112 | 13.5 | 2.1 | 81.8 | 6.9 | 0.6973 | -0.2079 | 0.1319 | **<0.0001** |
|  | Females | 128 | 12.2 | 1.7 | 80.2 | 6.0 | 0.5992 | -0.0575 | 0.0986 | **0.0003** |
|  | Both | 240 | 12.8 | 2.0 | 80.9 | 6.5 | 0.7123 | -0.2559 | 0.1278 | **<0.0001** |
| 4 | Males | 24 | 12.7 | 1.9 | 80.6 | 6.4 | 0.6009 | -0.0455 | 0.1049 | 0.1226 |
|  | Females | 30 | 11.5 | 1.8 | 79.2 | 6.1 | 0.4193 | 0.2617 | 0.0451 | 0.2601 |
|  | Both | 54 | 12.1 | 1.9 | 79.8 | 6.2 | 0.5662 | -0.0005 | 0.0794 | **0.0390** |
