# Supplementary material for: Vulnerability of a top marine predator to coastal storms: a relationship between hydrodynamic drivers and stranding rates of newborn pinnipeds
Source: Sci Rep. 2020 Jul 30;10:12807. doi: 10.1038/s41598-020-69124-6 (PMC7393492; doi:10.1038/s41598-020-69124-6)
Supplement: Supplementary file 4 — Supplementary file4 (DOCX 107 kb) [file 41598_2020_69124_MOESM4_ESM.docx]

**VULNERABILITY OF A TOP MARINE PREDATOR TO COASTAL STORMS: A RELATIONSHIP BETWEEN HYDRODYNAMIC DRIVERS AND STRANDING RATES OF NEWBORN PINNIPEDS**

Sepúlveda M.^1,2,3,*^, R. Quiñones^3,4^, C. Esparza^5^, P. Carrasco^3,4^ & P. Winckler^5,6,7^

^1^Centro de Investigación y Gestión de los Recursos Naturales (CIGREN), Universidad de Valparaíso, Valparaíso, Chile

^2^Núcleo Milenio de Salmónidos Invasores (INVASAL)

^3^Programa de Investigación Marina de Excelencia (PIMEX), Departamento de Oceanografía, Facultad de Ciencias Naturales y Oceanográficas, Casilla 160-C, Universidad de Concepción, Casilla 160-C, Concepción, Chile

^4^Interdisciplinary Center for Aquaculture Research (INCAR-FONDAP), Universidad de Concepción, O’Higgins 1695, Concepción 4070007, Chile

^5^Escuela de Ingeniería Civil Oceánica, Universidad de Valparaíso, Valparaíso, Chile

^6^Centro de Investigación para la Gestión Integrada del Riesgo de Desastres (CIGIDEN), Chile.

^7^Centro de Observación Marino para estudios de Riesgos del Ambiente Costero (COSTAR), Chile

*Corresponding author: Gran Bretaña 1111, Playa Ancha, Valparaíso, Chile. E-mail: [maritza.sepulveda@uv.cl](mailto:maritza.sepulveda@uv.cl). Phone: +56322508346

**SUPPLEMENTARY MATERIAL**

Table S2. Bivariate statistics for the JF period in the numerical node at 10 m depth offshore Cobquecura colony. Top: significant wave height and peak wave direction. Center: peak wave period and significant wave height. Bottom: and peak wave period and peak wave direction.

| Dpk vs Hm0 | | Hm0(m) | | | | | | | | | | A(%) | C(%) |
| --- | --- | --- | --- | --- | --- | --- | --- | --- | --- | --- | --- | --- | --- |
|  |  | < 0.5 | 0.5-1 | 1-1.5 | 1.5-2 | 2-2.5 | 2.5-3 | 3-3.5 | 3.5-4 | 4-4.5 | 4.5-5 |  |  |
| Dpk(ºN) | 225-240º |  | 0% | 0% | 0% | 0% |  |  |  |  |  | 0.7 | 100 |
|  | 240-255º |  | 0% | 6% | 18% | 12% | 2% | 0% | 0% | 0% |  | 38.6 | 99.3 |
|  | 255-270º |  | 0% | 4% | 12% | 14% | 8% | 2% | 0% | 0% | 0% | 39.5 | 60.8 |
|  | 270-285º |  | 0% | 1% | 1% | 0% | 0% | 0% | 0% | 0% |  | 1.9 | 21.2 |
|  | 285-300º |  | 0% | 5% | 10% | 4% | 0% | 0% | 0% |  |  | 19.4 | 19.4 |
|  | 300-315º |  |  |  |  | 0% | 0% | 0% |  |  |  | 0 | 0.0 |
|  | 315-330º |  |  | 0% | 0% |  |  |  |  |  |  | 0 | 0.0 |
|  | A(%) | 0.0 | 0.4 | 15.3 | 41.0 | 30.5 | 10.3 | 2.1 | 0.3 | 0.1 | 0.0 | 100.0 |  |
|  | C(%) | 100.0 | 100.0 | 99.6 | 84.2 | 43.2 | 12.8 | 2.4 | 0.4 | 0.1 | 0.0 |  |  |

| Hm0 vs Tp | | Tp(s) | | | | | | | | | | | | A(%) | C(%) |
| --- | --- | --- | --- | --- | --- | --- | --- | --- | --- | --- | --- | --- | --- | --- | --- |
|  |  | < 4 | 4-6 | 6.8 | 8-10 | 10-12 | 12-14 | 14-16 | 16-18 | 18-20 | 20-22 | 22-24 | >24 |  |  |
| Hm0(m) | < 0.5 |  |  |  |  |  |  |  |  |  |  |  |  | 0.0 | 100 |
|  | 0.5-1 |  |  |  |  | 0.1% | 0.1% | 0.1% | 0.1% | 0.1% | 0.0% | 0.0% |  | 0.5 | 100 |
|  | 1-1.5 |  |  | 0.2% | 0.3% | 3.6% | 5.1% | 2.5% | 1.9% | 1.1% | 0.4% | 0.1% | 0.1% | 15.3 | 99.5 |
|  | 1.5-2 |  |  | 0.3% | 1.3% | 5.3% | 19.2% | 4.5% | 5.9% | 3.1% | 1.0% | 0.4% | 0.1% | 41.1 | 84.2 |
|  | 2-2.5 |  |  | 0.0% | 1.5% | 1.4% | 16.7% | 4.5% | 3.3% | 2.0% | 0.8% | 0.2% | 0.1% | 30.5 | 43.1 |
|  | 2.5-3 |  |  |  | 0.3% | 0.3% | 5.8% | 2.3% | 1.0% | 0.3% | 0.2% | 0.0% | 0.0% | 10.2 | 12.6 |
|  | 3-3.5 |  |  |  | 0.1% | 0.0% | 0.8% | 0.6% | 0.5% | 0.1% | 0.1% | 0.0% |  | 2.2 | 2.4 |
|  | 3.5-4 |  |  |  |  |  | 0.0% | 0.1% | 0.1% | 0.0% |  | 0.0% |  | 0.2 | 0.4 |
|  | 4-4.5 |  |  |  |  |  |  |  | 0.1% | 0.0% |  |  |  | 0.1 | 0.1 |
|  | 4.5-5 |  |  |  |  |  |  |  | 0.0% |  |  |  |  | 0.0 | 0.0 |
|  | A(%) | 0.0 | 0.0 | 0.5 | 3.5 | 10.7 | 47.7 | 14.6 | 12.9 | 6.7 | 2.5 | 0.7 | 0.3 | 100.0 |  |
|  | C(%) | 100.0 | 100.0 | 100.0 | 99.5 | 96.0 | 85.3 | 37.6 | 23.0 | 10.1 | 3.4 | 0.9 | 0.2 |  |  |

| Dpk vs Tp | | Tp(s) | | | | | | | | | | | | | A(%) | C(%) |
| --- | --- | --- | --- | --- | --- | --- | --- | --- | --- | --- | --- | --- | --- | --- | --- | --- |
|  |  | < 4 | | 4-6 | 6.8 | 8-10 | 10-12 | 12-14 | 14-16 | 16-18 | 18-20 | 20-22 | 22-24 | >24 |  |  |
| Dpk(ºN) | 225-240º | |  |  | 0.5% | 0.0% | 0.1% |  |  |  |  |  |  |  | 0.7 | 100 |
|  | 240-255º | |  |  | 0.0% | 3.3% | 9.2% | 25.7% | 0.0% | 0.2% | 0.1% |  |  |  | 38.6 | 99.3 |
|  | 255-270º | |  |  | 0.0% | 0.1% | 1.1% | 21.5% | 11.0% | 4.7% | 0.7% | 0.3% | 0.0% | 0.0% | 39.5 | 60.8 |
|  | 270-285º | |  |  |  | 0.0% | 0.2% | 0.0% | 0.6% | 0.4% | 0.2% | 0.1% | 0.1% | 0.2% | 1.9 | 21.2 |
|  | 285-300º | |  |  |  | 0.0% | 0.0% | 0.4% | 2.9% | 7.6% | 5.6% | 2.0% | 0.7% | 0.1% | 19.4 | 19.4 |
|  | 300-315º | |  |  |  | 0.0% | 0.0% | 0.0% |  |  |  |  |  |  | 0.0 | 0.0 |
|  | 315-330º | |  |  |  |  |  |  |  |  |  |  |  |  | 0.0 | 0.0 |
|  | A(%) | 0.0 | | 0.0 | 0.5 | 3.4 | 10.6 | 47.7 | 14.7 | 12.9 | 6.6 | 2.4 | 0.8 | 0.3 | 100 |  |
|  | C(%) | 100.0 | | 100.0 | 100.0 | 99.5 | 96.0 | 85.5 | 37.8 | 23.1 | 10.2 | 3.6 | 1.2 | 0.3 |  |  |
